# Age at Which Larvae Are Orphaned Determines Their Development into Typical or Rebel Workers in the Honeybee (*Apis mellifera* L.)

**DOI:** 10.1371/journal.pone.0123404

**Published:** 2015-04-16

**Authors:** Karolina Kuszewska, Michal Woyciechowski

**Affiliations:** Institute of Environmental Sciences, Jagiellonian University, Krakow, Poland; University of North Carolina, Greensboro, UNITED STATES

## Abstract

In the honeybee, diploid larvae fed with royal jelly develop into reproductive queens, whereas larvae fed with royal jelly for three days only and subsequently with honey and pollen develop into facultatively sterile workers. A recent study showed that worker larvae fed in a queenless colony develop into another female polyphenic form: rebel workers. These rebel workers are more queenlike and have greater reproductive potential than normal workers. However, it was unclear whether larvae orphaned at any time during their feeding period can develop into rebels. To answer this question, the anatomical features of newly emerged workers reared in queenless conditions at different ages during the larval period were evaluated. Our results showed that larvae orphaned during the final four or more days of their feeding life develop into rebel workers with more ovarioles in their ovaries, smaller hypopharyngeal glands, and larger mandibular and Dufour’s glands compared with typical workers with low reproductive potential that were reared with a queen or orphaned at the third to last or a later day of feeding life.

## Introduction

An important topic in modern biology is how organisms modify their life strategy (including their physiology, morphology and anatomy) depending on the environmental conditions where they live or lived during a certain period of their life [1]. Of particular interest is how one genotype is able to produce two or more distinct phenotypes. This phenomenon, referred to as polyphenism, is widespread among many organisms [2–4]. Polyphenism is the major reason for the success of insects [4], as it allows them to partition life history stages from the larva and pupa to the adult stage and to adopt different phenotypes during seasonal environmental conditions or upon unpredictable environmental changes, e.g., degradation due to overcrowding. The most familiar and spectacular examples of polyphenism, based primarily on the reproductive division of labour, are provided by eusocial insects [5]. The reproductive and non-reproductive castes, i.e., the queens and workers, typically exhibit divergent morphologies as well as behaviour and physiology [6].

Among eusocial insects, the process underlying honeybee (*Apis mellifera*) caste polymorphisms is the best understood [7–10]. The expression of the queen or worker phenotype depends mainly on larval nutrition. Larvae fed with relatively small amounts of royal jelly by nurse bees develop into workers, which are small in size and facultatively sterile, whereas larvae fed with large amounts of royal jelly develop into queens [11], which are large in size and have an active reproductive system. The quality and quantity of food provided at larval stages affect DNA methylation [12,13], gene expression [14,15], juvenile hormone titres [16,17] and the haemolymph protein composition [18–20]. In the honeybee, the differences between female castes and sub-castes (i.e., the queen, workers, rebel workers) are determined during a 6-day period of feeding larval development (unsealed larva), which follows a 3-day period of egg incubation and precedes the sealed larva, prepupal, pupal (metamorphosis) and adult stages (development from an egg to adult takes 21 days). In some studies, unsealed larvae are considered to be bipotential up to the third day of life [7,21,22] and able grow into either queens or workers, depending on epigenetics. By contrast, several papers have provided evidence that 3-day-old unsealed larvae that subsequently develop into queens or workers already show differences in protein expression levels [19,20].

A recent study demonstrated that in the honeybee, there is another sub-caste of females—the rebel workers [23]. These rebels develop immediately after swarming, which is the only natural means of colony multiplication, and they exhibit significantly more ovarioles in their ovaries as well as more developed mandibular glands than the queen, in addition to underdeveloped hypopharyngeal glands. These features suggest that rebels are more engaged in laying their own male-determined eggs than in rearing the queen’s offspring. A previous study confirmed this suggestion, as 15-days old rebel workers displayed active ovaries if they remained in a queenless or a queenright colony during their adult life [23]. The appearance of workers with mature ovaries in orphaned colonies is not surprising. Workers are known to lay eggs if a colony loses its queen and there is no chance to rear a new queen [24,25]. However, the readiness of rebels to reproduce in queenright colonies is more unexpected because of the hypothesis that the presence of a queen effectively inhibits workers’ oogenesis [24,25]. It has been suggested that the proximate factor that influences rebel sub-caste development is the absence of a queen during the larval feeding period (unsealed larva), whereas the decreased relatedness between the old queen’s workers and the new queen’s offspring appears to be the ultimate factor justifying the shift in resource reallocation to reproductive tissue in rebels [23]. This finding provides opportunities to investigate issues related to the evolution of sterile workers and polyphenism and the factors that have been important in this evolutionary process from a different perspective [26].

The aim of the present study was to determine the age at which honeybee worker larvae are able to divert their life strategy after orphaning by their colony and instead develop into rebel workers. For this purpose, honeybee colonies containing larvae of different ages were reared in queenless conditions (orphaned), and newly emerged workers were weighed and dissected to evaluate their development. We predicted that larvae orphaned at a younger age would be more likely to develop into rebel workers, whereas those orphaned at an older age would develop into normal, non-rebel workers.

## Materials and Methods

**Fig 1:**
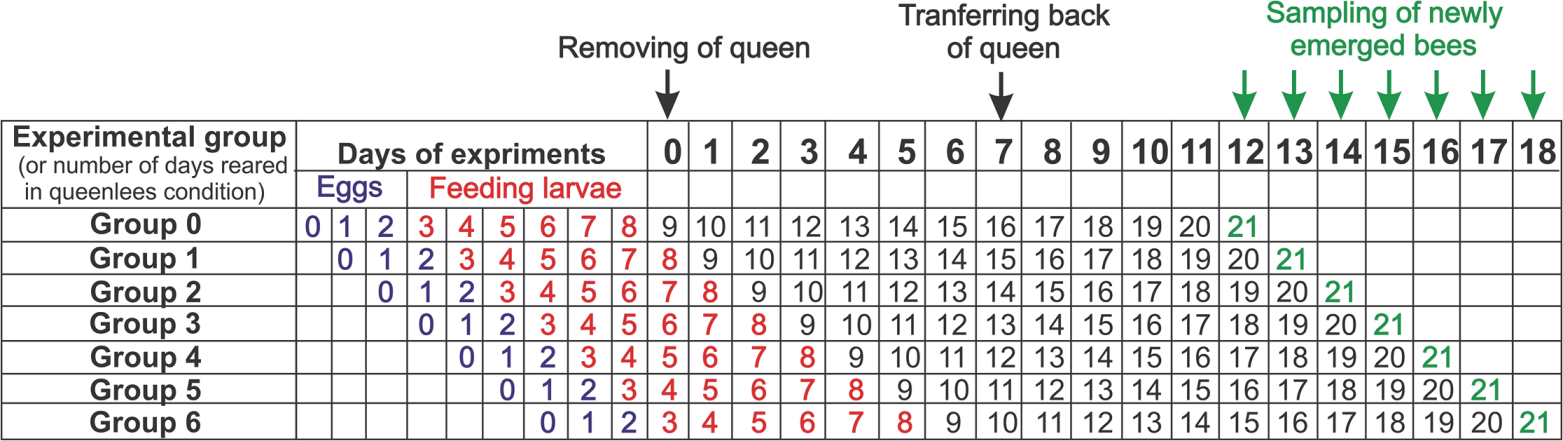
Time schedule of manipulations and the experimental days when workers were sampled from the seven groups (indicated in green). The time of egg incubation is indicated in blue; feeding larvae (unsealed larvae) are indicated in red; and sealed larvae or pupae are indicated in black.

The research was conducted in July 2010 in the experimental apiary of the Institute of Environmental Sciences (Jagiellonian University, Krakow, southern Poland). Five queenright honeybee (*A*. *m*. *carnica*) colonies were studied, each consisting of 20 000–40 000 workers. All colonies were treated in the same way, as shown in Fig 1. Before the experiment began, three empty frames of wax combs were placed in each experimental colony to increase the area available for eggs laying by the queen. At the beginning of the experiment, each colony was temporarily orphaned (day 0), and each queen was transferred to a new hive box with a small group of workers. Workers, pupae, sealed larvae and unsealed larvae of different ages remained in the native queenless colonies. One week after orphaning (day 7), the queens were transferred back to their native colonies. As a result of this manipulation, larvae were reared under seven different conditions (groups 0–6; Fig 1) inside each native colony. The larvae of group 0 were raised in the queenright condition during their entire feeding period, before the colony was orphaned. The larvae from groups 1 to 5 were raised in the queenless condition for the last 1 to 5 days of their feeding period, respectively. Larvae of group 6 hatched from eggs after the queen was transferred to the new hive box, such that they were reared in the queenless condition during their whole feeding period. On day 12 of the experiment, the frames with sealed broods were transferred from each colony to the laboratory, and 30 newly emerged workers (reared as larvae in the queenright condition—group 0) were sampled. This procedure was repeated for 7 consecutive days (from day 12 to 18 of the experiment; Fig 1) to sample the workers reared as larvae without the queen for different numbers of days (groups 0–6; all workers from all experimental groups emerged in an incubator in the laboratory). All of these workers were weighed and killed by freezing (–16°C) and subsequently dissected under a stereomicroscope (binocular loupe). The number of ovarioles and the sizes of the hypopharyngeal, mandibular and Dufour’s glands were determined to discriminate rebel and non-rebel works [23]. In honeybees, the number of ovarioles is a good indicator of the reproductive potential of females [27,28], and the mandibular and Dufour’s glands are usually larger in queens and reproductive workers than in non-reproductive workers [11]. The size of the hypopharyngeal glands, which synthesize and store brood food, depends on the social status of workers [29,30] and are largest in nursing bees [31]. The total number of ovarioles in both ovaries of each worker was recorded. The size of the hypopharyngeal glands was calculated from the average of 10 acini (each acinus was measure as the square root of the longest × shortest diameter, and the average was calculated from 5 acini from the right gland and 5 from the left gland). The hypopharyngeal gland consists of a great number of lobes, called acini, and their diameter is routinely used as an index of gland size [23,32–34]. The size of the mandibular gland was calculated from the average of the left and right glands (each gland was measure as the square root of the longest × shortest diameter). The size of Dufour’s gland was also calculated as the square root of the longest × shortest diameters. All organs were stained with Giemsa reagent (approximately 10 seconds) before measured.

Mixed model two-way ANOVA was used to compare parameters (body mass, ovariole number, hypopharyngeal gland size, mandibular gland size, and Dufour’s gland size) between the 7 groups of workers, with experimental group as a fixed effect and colony as a random effect. If an experimental group was statistically significant, ANOVA was followed by multiple comparisons using the post hoc Tukey HSD test with *P* = 0.05 considered significant. The total ontogeny of the workers reared in different conditions (experimental groups) was analysed using principal component analysis (PCA).

The PCA test reduced the total set of partly intercorrelated variables to two uncorrelated principal components (PC1 and PC2). This analysis was performed using the average values of body mass, ovariole number, hypopharyngeal gland size, mandibular gland size, and Dufour’s gland size for groups of bees from each experimental group (7 groups) and each colony (5 colonies) (a total of 35 measurement points). Next, two ANOVAs with Tukey tests for multiple comparisons of means were performed. In these analyses, factor coordinates for the 35 groups of workers (coordinator of cases for PC1 and PC2; S1 Table), which were calculated in the PCA, were used. All calculations were performed with STATISTICA 9.0.

## Results

**Fig 2:**
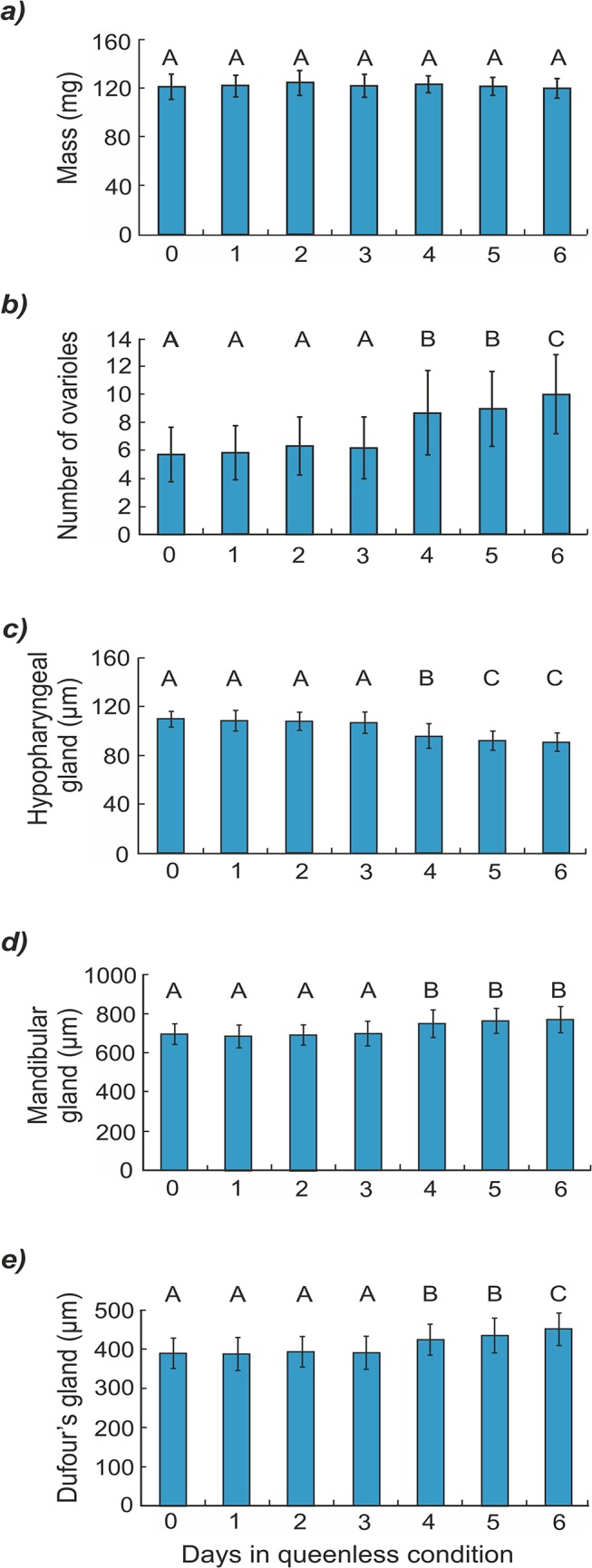
Mean (± SD) anatomical parameters for bees reared as larvae under seven different conditions. Body mass **(a)** number of ovarioles **(b)** size of hypopharyngeal glands **(c)** size of mandibular glands **(d)** and size of Dufour’s gland **(e)** of newly emerged honeybee workers reared as larvae in the queenless condition for 0 to 6 days (see Methods; each bar represents pooled data from five colonies; groups that differ significantly from one another are indicated with different letters).

Our results show that newly emerged workers reared as larvae without the queen at different stages of development did not differ with respect to body mass (Fig 2A and Table 1). However, there were significant differences in ovariole numbers and the size of hypopharyngeal, mandibular and Dufour’s glands between workers from different experimental rearing groups (Table 1 and Fig 2 B–2 E). The post hoc Tukey HSD test showed that workers reared in the queenright condition (group 0) and those reared in the queenless condition for the last 1, 2 or 3 days of the larval feeding period (groups 1–3) exhibited fewer ovarioles, larger hypopharyngeal glands and smaller mandibular and Dufour’s glands than the workers reared in queenless conditions for the last 4, 5 or 6 days of the larval period (groups 4–6; Fig 2; *P* < 0.001). There were also differences in some parameters (the number of ovarioles and the size of hypopharyngeal and Dufour’s glands) between the workers from groups 4–6 (details in Fig 2 B–2 E; P < 0.001).

**Table 1:** Results of mixed model two-way ANOVA for body parameters of newly emerged honeybee workers reared as larvae for 0 to 6 days in the queenless condition (7 experimental groups—fixed factor) and different colonies (random factor).

Parameters	Factors	*d*.*f*.	*F*	*P*
Mass	Groups (G)	6	*F*_*6*,*24*_ = 1.3	0.284
	Colony (C)	4	*F*_*4*,*1011*_ = 297.2	< 0.001
	G*C	24	*F*_*24*,*1011*_ = 7.4	< 0.001
Number of ovarioles	Groups (G)	6	*F*_*6*,*24*_ = 162.0	< 0.001
	Colony (C)	4	*F*_*4*,*1011*_ = 24.4	< 0.001
	G*C	24	*F*_*24*,*1011*_ = 0.5	0.966
Hypopharyngeal glands	Groups (G)	6	*F*_*6*,*24*_ = 128.4	< 0.001
	Colony (C)	4	*F*_*4*,*1011*_ = 4.5	0.001
	G*C	24	*F*_*24*,*1011*_ = 1.3	0.165
Mandibular glands	Groups (G)	6	*F*_*6*,*24*_ = 86.0	< 0.001
	Colony (C)	4	*F*_*4*,*1011*_ = 1.8	0.124
	G*C	24	*F*_*24*,*1011*_ = 0.7	0.898
Dufour’s gland	Groups (G)	6	*F*_*6*,*24*_ = 66.5	< 0.001
	Colony (C)	4	*F*_*4*,*1011*_ = 12.2	< 0.001
	G*C	24	*F*_*24*,*1011*_ = 1.0	0.521

**Fig 3:**
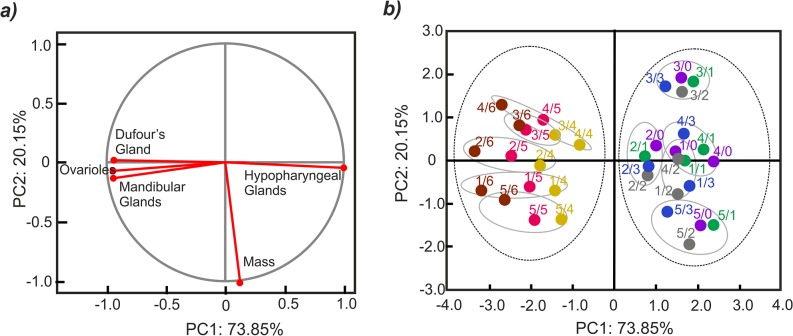
Results of PCA based on the average values of workers’ ontogenic parameters (body mass, ovariole number, hypopharyngeal gland size, mandibular gland size and Dufour’s gland size). Biplot **(a)** with loading variables showing the correlations of the environmental variables with PC1 and PC2. In the score plot **(b)** the different colours and numbers describe groups of workers originating from different colonies, reared for different numbers of days in the queenless condition (e.g. number 4/3 indicates workers that originated from the fourth colony and group 3, whereas number 2/0 indicates workers originating from the second colony and group 0). The dotted circles indicate two clusters of workers (rebel and non-rebel), whereas the grey circles indicate sub-clusters of workers from each colony based on their similarity in body mass.

In the PCA, the two principals (PC1 and PC2) account for 94.00% of the variance between the groups of workers reared as larvae in different conditions and colonies (Table 2). The correlations of the environmental variables with the PC1 and PC2 axes are given as vectors in the biplot (Fig 3A). The first axis (PC1) accounts for 73.85% of the variance and is negatively correlated with ovariole number and the size of the mandibular and Dufour’s glands and positively correlated with hypopharyngeal gland size (Fig 3A). The second axis (PC2) accounts for 20.15% of the variance (Table 2) and is negatively correlated with the body mass of the workers (Fig 3A). The PCA reveals two very conspicuous clusters of workers, based on their similarities in anatomical parameters (PC1). The first cluster (Fig 3B) consists of workers reared as larvae in the queenright condition (group 0) and those reared for the last 1, 2 or 3 days of their feeding period in the queenless condition (groups 1–3). The second cluster, by contrast, is composed of workers reared in the queenless condition for the last 4, 5 or 6 days of the larval feeding period (Fig 3B). In each of these two clusters, we can also designate sub-clusters based on similarities in body mass (PC2). These sub-clusters consist of groups of workers originating from each colony (Fig 3B). Finally, two ANOVAs with Tukey tests were performed using factor coordinates for the 35 groups of workers (cases) that were calculated in the PCA. In the first analysis, the coordinates described by the PC1 axis were used. These results confirmed that workers from groups 0–3 developed into normal workers, whereas those from groups 4–6 developed into rebel individuals (*P* < 0.001). The analysis also indicated that workers from groups 4–6 differ from one another (groups 4 and 5, *P* = 0.004; groups 4 and 6, *P* < 0.001; groups 5 and 6, *P* = 0.001). Moreover, anatomical parameters also depend on the colony (*P* < 0.001). The coordinates described by the PC2 axis were used in the second analysis. Here, the results showed that body mass depends on the workers’ colony of origin (*P* < 0.001) and that the workers originating from the second colony had a lower body mass than those originating from other colonies. However, experimental group had no significant effect on body mass (*P* = 0.409).

**Table 2:** Principal component loadings of the measured ontogeny of workers reared in different conditions.

	Rotated component loadings	
Variable	PC1	PC2
Mass	0.1188	**-0.9922**
Number of ovarioles	**-0.9558**	-0.0651
Size of hypopharyngeal glands	**0.9808**	-0.0434
Size of mandibular glands	**-0.9513**	-0.1269
Size of Dufour’s glands	**-0.9475**	0.0237
Variance explained by rotated components	73.85%	20.15%

Loadings given in boldface show the highest correlation between the original values (average values for groups of bees from each experimental group and each colony) and principal component scores.

## Discussion

We examined how the absence of a queen at different larval ages affects the life strategy of developing workers. A previous study showed that worker larvae reared in queenright conditions develop into normal workers, whereas those reared without the queen develop into rebel workers [23]. However, it was not clear how late in development a larva can change its life strategy. In this report, we show that only larvae orphaned before the third day of their life (groups 4–6) exhibit the possibility to develop into individuals that are anatomically similar to rebel workers [23], whereas those that are orphaned at an older age (3 days old or older; groups 0–3) developed into normal workers (Fig 3B). These data are in accordance with studies demonstrating differences in protein expression between worker and queen castes during early developmental larval stages, less than 72 hours (3 days) after hatching from eggs [19,20]. On the other hand, other studies have shown that 3-day-old larvae are still bipotential and can develop into both workers and queens depending on feeding conditions [7,21,22], which has also been confirmed by beekeeper practices. It should be noted that all of these earlier studies focused on queen/worker caste determination. Accordingly, larvae (including 3-day-old individuals) were transferred from worker’s cells to large queen’s cups, where nursing workers can provide the larvae with much more food. Moreover, the queen’s cells exhibit a different spatial orientation (vertically oriented) than the worker’s cells (horizontally oriented), which also affects queen/worker caste determination [30]. In our experiment, we focused on worker caste determination, and the tested larvae were reared in worker’s cells during the entire experimental period. In this case, the signal that triggered a more selfish strategy was more closely associated with the absence of the queen than with the amount and quality of food or the size and orientation of the larva’s cell. In this regard, we believe that the factor that triggers the larva’s decision depends on whether it is a question of queen/worker or normal/rebel worker caste determination.

We found that some larvae were too old to change their anatomical parameters. This may mean that the larvae are sensitive to some environmental cues important to their caste determination only during a certain period in their life. It is well known that many insect species have a critical period when individuals are sensitive to inductive stimuli. This critical period often occurs long before the alternative phenotype actually develops [3]. For example, a different photoperiod between 28 and 48 hours after pupation leads to wing morph determination in the butterfly *Precis coenia* [35], and heat shocks during the fourth larval period instar lead to fifth instar larvae determination of colours in the moth *Manducta sexta* [36]. In our experiment, an alternative explanation is that the larvae are sensitive to the absence of the queen during all larval stages but do not always have enough time to change the previously selected life strategy. Our investigation seems to confirm this second interpretation because we found a significant difference not only between rebel and normal workers but also between the groups of rebel workers that were reared as larvae in the queenless condition for differing numbers of days. The larvae that spent a shorter time in the nest without the queen (groups 4 and 5) had less developed rebel traits than those that were reared in an orphaned colony for the whole feeding period (group 6; Figs 2 and 3B). This pattern suggests that these individuals did not manage to carry out all of the changes in their body because they did not have enough time.

Some authors have proposed that all larvae in orphaned colonies are fed more often [37] and receive larger amounts of food [38] than those reared in colonies with a queen. There are also studies showing that the body mass of newly emerged workers originating from colonies selected for high or low pollen hoarding is correlated with the number of ovarioles [39,40]. Because of these findings, we assessed whether the body mass of adult workers depended on how much time they spent in queenless conditions during their larval feeding period. The results showed that freshly emerged workers did not differ in body mass, regardless of how long they were fed in the orphaned colony (Fig 2A and Table 1). This finding is consistent with a previous report showing no difference in body mass between rebel and normal workers [23] and may suggest that larvae reared in queenright and queenless conditions are fed with similar amounts of food. On the other hand, a study by Hoover and colleagues [41] showed that 10-day-old adult honeybee workers reared as larvae with a high-quality diet exhibited better-developed ovaries but did not differ in body mass in comparison with workers reared as larvae with a low-quality diet. However, this result could have been due to the fact that the workers’ mass was measured on the 10^th^ day of their life. Thus, the workers could have compensated for any effect of the low-protein larval diet in their adult life. In the present study, we did not measure the feeding rates or quality of provided food, and for this reason, we cannot exclude the possibility that any of these factors affected the switch to a more queen-like physiology in larvae reared in queenless conditions. Additionally, our analysis showed that body mass is not a random element but depends on the origin of workers (Fig 3B). In other words, the colony of origin had a greater impact on the workers’ body mass than the experimental treatment.

The rebel worker strategy is associated with certain changes in anatomical parameters. In this work, we examined the size of Dufour’s gland for the first time, which is generally more developed in the honeybee queen than in workers. The chemical composition of Dufour’s gland is also affected by the caste, task and age of the bees [42,43]. The function of Dufour’s gland remains somewhat controversial. Some reports have suggested that it produces egg-marking pheromones [44,45], although another investigation showed that this is unlikely because the eggs do not pass directly over the exit of Dufour’s gland [46]. It is currently believed that Dufour’s gland produces chemical substances that inform the colony members about the reproductive state of the queen [47–49] and dominant workers, which are more likely to ultimately become reproductive individuals producing queen-like blends of Dufour’s gland extract [50–52]. Our results show that Dufour’s gland was larger in workers reared in queenless conditions than in those reared with a queen (Fig 2D). This finding is in accordance with studies suggesting a connection between gland activity and gland size [52] and confirms that newly emerged rebel workers invest more in their own reproduction than newly emerged non-rebel workers [23].

An interesting issue is how worker larvae are able to sense the absence of the colony’s queen. It is possible that honeybee larvae can detect the lack of queen mandibular gland pheromones (QMPs), similar to adult workers [53,54]. There is no evidence of such an ability in honeybees; however, it was shown that in the bumblebee (*Bombus terrestris*), caste differentiation during the larval stage may be determined by the queen’s pheromones [55]. It is also known that a lack of brood pheromones can influence the physiology and behaviour of workers [56–58]. In a previous study [23], it was shown that brood pheromones likely have no impact on rebel/normal worker caste determination, as both of these sub-castes of workers were reared in colonies without a brood [23]. We also cannot exclude the possibility that larvae receive a better quality and quantity of food if they are reared in queenless conditions, as discussed above.

To summarise, our study determined the age at which larvae are capable of changing their life strategy to become rebel workers. Based on the anatomical traits of honeybee workers, we report that this strategy is possible only in those individuals that spend more than four days of their larval life in an orphaned colony. Our findings also indicate that there are likely some differences in the mechanisms underlying queen/worker and rebel/normal worker caste determination. Further studies should aim to achieve a better understanding of both of these mechanisms and the evolution of polyphenism.

## Supporting Information

S1 Table Factor coordinates of cases used in ANOVAs based on correlations in the PCA analysis for PC1 and PC2. Each number describes the coordinates for workers from each colony and experimental treatment. (PDF)
