# Supplementary material for: Age at Which Larvae Are Orphaned Determines Their Development into Typical or Rebel Workers in the Honeybee (Apis mellifera L.)
Source: PLoS One. 2015 Apr 16;10(4):e0123404. doi: 10.1371/journal.pone.0123404 (PMC4400021; doi:10.1371/journal.pone.0123404)
Supplement: S1 Table — (PDF) [file pone.0123404.s001.pdf]

Days without queen	Colonies	PC1	PC2
0	1	1.4639	0.20874
0	2	0.99214	0.33959
0	3	1.58877	1.91183
0	4	2.37196	-0.0284
0	5	2.06744	-1.51556
1	1	1.67903	0.00922
1	2	0.7203	0.11054
1	3	1.87264	1.84481
1	4	2.13291	0.26275
1	5	2.38413	-1.49184
2	1	1.50745	-0.78109
2	2	0.79709	-0.34086
2	3	1.62392	1.60696
2	4	1.52047	0.0534
2	5	1.80081	-1.94148
3	1	1.78867	-0.5877
3	2	0.81584	-0.13844
3	3	1.20453	1.73348
3	4	1.66434	0.61511
3	5	1.26233	-1.17796
4	1	-1.43006	-0.68465
4	2	-1.79276	-0.10698
4	3	-1.43948	0.60844
4	4	-0.844	0.35606
4	5	-1.28142	-1.36475
5	1	-2.04596	-0.5988
5	2	-2.49326	0.1114
5	3	-2.12531	0.70461
5	4	-1.71804	0.94792
5	5	-1.92237	-1.39119
6	1	-3.19411	-0.7005
6	2	-3.35003	0.21724
6	3	-2.28468	0.81963
6	4	-2.70444	1.28567
6	5	-2.63275	-0.89721
